# UPLC–MS-Based Non-targeted Analysis of Endogenous Metabolite Changes in the Leaves of *Scabiosa tschiliensis* Grüning Induced by 6-Benzylaminopurine and Kinetin

**DOI:** 10.3389/fpls.2021.700623

**Published:** 2021-07-21

**Authors:** Jialin Du, Weiwei Ma, Yi Li, Xu Lu, Zhaopeng Geng, Hangjun Huang, Yuanyuan Yuan, Yue Liu, Xiaodong Wang, Junli Wang

**Affiliations:** ^1^Key Laboratory of Ecology and Environment in Minority Areas (Minzu University of China), National Ethnic Affairs Commission, Beijing, China; ^2^College of Life and Environmental Sciences, Minzu University of China, Beijing, China

**Keywords:** *S. tschiliensis*, plant growth regulator, adventitious shoot, metabolic pathways, metabolomics

## Abstract

*In vitro* propagation technology with plant growth regulators (PGRs) is generally applied in the cultivation of *Scabiosa tschiliensis*, which can solve collection difficulties and limited resources of *S. tschiliensis*. Nevertheless, comprehensive metabolomic evaluation on *S. tschiliensis* with PGR effects is still lacking. In this work, a non-targeted metabolomics approach, coupled with statistical and pathway enrichment analysis, was used to assess the regulatory influences of 6-benzylaminopurine (6-BA) and kinetin (KT) applied in *S. tschiliensis*. The results showed that the PGRs affect metabolism differentially, and the addition of 6-BA and KT can increase different secondary metabolites. In the two PGR groups, some primary metabolites such as L-phenylalanine, L-tyrosine, L-arginine, L-asparagine, and D-proline were significantly reduced. We suspect that under the action of PGRs, these decreased amino acids are derived into secondary metabolites such as umbelliferone, chlorogenic acid, and glutathione. Additionally, some of those secondary metabolites have a biological activity and can also promote the plant growth. Our results provide a basis for the targeted cultivation and utilization of *S. tschiliensis*, especially the expression of metabolites related to PGR application.

## Introduction

*Scabiosa tschiliensis* Grüning belonging to the Dipsacaceae family is a perennial herb. The flower is valuable for both its delightful ornamental properties and its chemical compositions, including flavonoids, triterpenes, coumarins, and other metabolites (Wang and Xue, 2009; Ma et al., 2016). These metabolites are significant in the development and application of *S. tschiliensis*, as the pharmacological functions are closely related to them. *S. tschiliensis* has been reported with the functions of antipyretic, anti-inflammation, and antioxidant, and it can enhance the immune and cardiovascular systems. The flowers have a long history of medicinal use in the treatment of fever, headache, coughing, liver toxicity, jaundice, and other diseases in Inner Mongolia of China (Wang et al., 2013). However, *S. tschiliensis* is distributed at higher elevations (300–1,500 msl), and the resources are very limited. Moreover, due to the extensive use of flowers as medicine materials, seed production is reduced. Conventional seed reproduction cannot ensure a large-scale production because it is difficult to collect seeds. Based on the above problems, and in consideration of increasing demands for *S. tschiliensis*, a new approach is needed for mass propagation (Wang et al., 2013).

*In vitro* propagation technology provides a powerful tool that may rapidly add the number of plants as well as propagules and can even aid to replace natural populations (Guo et al., 2007; Acemi et al., 2020). Plant growth regulators (PGRs), such as 6-benzylaminopurine (6-BA) and kinetin (KT), have been served as versed elicitors to stimulate the production of secondary metabolites in the cultivation of medicinal plants (Jamwal et al., 2018; Acemi, 2020). For example, compared with the petioles of unchanged plant in turmeric, the level of berberine in callus cultured with PGR was approximately 18 times higher (Khan et al., 2008); the presence of PGRs accumulated a plenty of flavonoids in comparison with natural plants in *Hypericum mysorense* (Shilpashree and Rai, 2009). The use of tissue culture technology to propagate *S. tschiliensis* can provide sources for further expansion of cultivation and obtain its medicinal ingredients in the future. Induced effects of different PGRs on the *in vitro* culture of *S. tschiliensis* have been studied, and a system of rapid micropropagation has been found (Wang et al., 2013). However, an overall metabolic evaluation of the effect of PGRs in *S. tschiliensis* is lacking.

Metabolomics is particularly interesting since it can reveal a global assessment of the relative levels of metabolites from hundreds to thousands in the material being studied (Van Meulebroek et al., 2012). Ten years on, the approach has developed into a promising technology that can assess the overall impact and potential mechanisms of natural product research under specific conditions (An et al., 2018). As a consequence, metabolomics has the potential to decipher the influence of a certain PGR on metabolites.

High-throughput techniques are usually used to profile these large numbers of unknown metabolites highly diverse in physical and chemical properties. Additionally, statistical processing of complex datasets is required to recognize and characterize the metabolites (Jandrić et al., 2014). At present, a lot of methods are available in metabolomics, such as non-destructive nuclear magnetic resonance spectroscopy (NMR) (Kang et al., 2008; Wen et al., 2010), gas chromatography–mass spectrometry (GC–MS) (Khakimov et al., 2014), and Fourier transform–ion cyclotron resonance–mass spectrometry (FI–ICR–MS) (Takahashi et al., 2008). Recently, ultra-high-performance liquid chromatography coupled to mass spectrometry (UPLC–MS) has been evolved to decipher bioactive compounds from complex mixtures due to the high reliability, high sensitivity, fast speed, and easy operation (Zhang et al., 2019). The advantages of this technique make it an ideal tool to detect metabolites and evaluate their possible mechanisms (Allwood and Goodacre, 2010).

This work aimed to apply a non-targeted metabolomic strategy, based on UPLC–MS with multivariate statistical analysis, to evaluate the two different PGRs, namely, 6-BA and KT, on *S. tschiliensis*. The differential metabolites following different PGRs were also investigated to discover the potential regulatory mechanisms of the two PGRs.

## Materials and Methods

### Plant Materials

*In vitro* production of plant material. Seeds of *S. tschiliensis* were collected from Lingshan Mountain (40°1′51″ north latitude, 115°27′19″ east longitude, 2,100–2,330 msl) in the Beijing area of China, from September 2018. The seeds were washed and the outer seed coat was peeled off; the surface was disinfected with 75% alcohol for 30 s and 12% (v/v) H_2_O_2_ for 20 min. Traces of H_2_O_2_ were rinsed three times with distilled sterile water. These seeds were aseptically inoculated on the growth regulator-free MS (Murashige and Skoog 1962) to obtain shoots. The shoots from *in vitro* germinated seeds were divided into two parts: One part was used to induce adventitious shoots, and the other part was cultivated for 45 days as plant materials for the control group (Supplementary Figure 1).

Induction of adventitious shoots. When the shoots from *in vitro* germinated seeds were cultivated for 30 days, healthy ones with a height of about 4 cm were selected as explant donors, and leaves were collected as explants. Due to the high induction frequency of PGRs at 4.0 mg L^−1^ (Supplementary Table 1), the leaves (0.5 × 0.5 cm in size) were cultured on a semisolid MS medium added with 4.0 mg L^−1^ 6-BA and 4.0 mg L^−1^ KT to induce adventitious shoots, respectively. Three repetitions were operated on the experiments, with 30 explants for each treatment. One day after implantation *in vitro*, the adventitious shoots were removed from the callus and transferred to a fresh medium with corresponding PGR. After the explants were cultured for 45 days, the plant leaves were collected and stored at −80°C (Supplementary Figure 1).

All MS media were added with 30 g L^−1^ sucrose and 7 g L^−1^ of plant agar (Biotopped, Beijing, China). Culture media pH was adjusted to 5.7 with 1 mol L^−1^ NaOH or 1 mol L^−1^ HCl prior to autoclaving. Shoots from *in vitro* germinated seeds and adventitious shoots induced by PGRs were grown in a sterile culture room in a controlled environment (24 ± 1°C, 70% relative humidity, 30–40 μmol m^−2^ s^−1^ light intensity, and 12-h light/dark cycles). Experimental materials were divided into three experimental treatment groups: leaves of shoots from *in vitro* germinated seeds (control group), leaves of adventitious shoot induced by 6-BA, and leaves of adventitious shoot induced by KT.

### Chemicals

Acetonitrile, methanol, and formic acid of LC/MS grade were the products of Sigma-Aldrich (St. Louis, MO, United States). Ultrapure water used throughout the experiments was supplied by a Milli-Q system (Millipore, United States).

### Sample Preparation

Approximately 300 mg of the leaves was used for the preparation of tissue homogenates; three porcelain beads and 500 μL extract solution (methanol: water = 4: 1) were added. Then, the samples were homogenized at 7,500 × g for 30 s at 2°C, and the homogenization cycle was repeated two times. Then the samples were centrifuged at 15,000 × *g* for 4 min at 2°C. The supernatant was filtered using a 0.22-μm filter membrane and transferred to a glass vial for analysis.

### Chromatographic Analysis

Chromatographic separation was performed on a UPLC (Dionex UltiMate 3000, Thermo Fisher Scientific, Waltham, United States) adopted to an Acquity UPLC® BEH C18 column (Waters, United States, 1.7 μm, 2.1 × 50 mm). The mobile phase was A (water with 0.1% formic acid, v:v) and B (acetonitrile with 0.1% formic acid, v:v) with a flow rate of 0.3 mL min^−1^, and the gradient was as follows: 0–30 min, 5–100% B; 30–35 min, 100% B; 35–40 min, 5% B. The column temperature was maintained at 25°C, and the injection volume was 10 μL.

High-resolution mass spectrometer system of impact II Q–TOF–MS/MS (Bruker Daltonics Corporation, Germany) coupled with an electrospray ionization source was used for experiments. The drying gas flow rate was set to 8.0 L min^−1^, and the temperature was 220°C. The ionization source was adjusted to the positive mode, and the spray voltage was 4,500 V. Data were collected in the range from *m/z* 50–1,300 with the five strongest ions that were selected for automatic lysis (Auto-MS/MS).

### Data Processing and Multivariate Pattern Analysis

The Bruker FlexAnalysis 3.4 software was used to perform UPLC–MS data, and multivariate analysis was dealt with MetaboAnalyst 5.0 platform (https://www.metaboanalyst.ca) and SIMCA software 14.0, including principal component analysis (PCA) as well as orthogonal partial least-squares discriminant analysis (OPLS-DA). PCA was initially applied to identify the differences between the sample groups (Chen et al., 2009). Then the OPLS-DA was performed to identify the metabolites that cause group differences, and the potential metabolites were obtained through VIP of OPLS-DA. To evaluate the OPLS-DA model, model fitness (*R*^2^) and predictive ability (*Q*^2^) were used, and the permutation tests (200 times) can be used for further verification (Cloarec et al., 2005). The differential metabolites were subjected to further MS/MS identification of the molecular formula with the help of available public metabolome databases, such as METLIN (http://metlin.scripps.edu) and Human Metabolome Database (HMDB, http://www.hmdb.ca). Three parent ion adducts of [M + H]^+^, [M + Na]^+^, and [M + H–H_2_O]^+^ were considered in the database search, and the allowable mass error was ±10 ppm.

### Metabolic Pathway Analysis

The metabolic pathways were analyzed to evaluate the impact of the potential differential metabolites on cell metabolism, and the visualization was done by MetaboAnalyst (http://metpa.metabolomics.ca) (Xia and Wishart, 2010), which is mainly based on HMDB and the Kyoto Encyclopedia of Genes and Genomes (KEGG) pathway database (http://www.kegg.jp/kegg/pathway.html).

## Results

### Detection of Metabolites According to UPLC–MS

**Figure 1:**
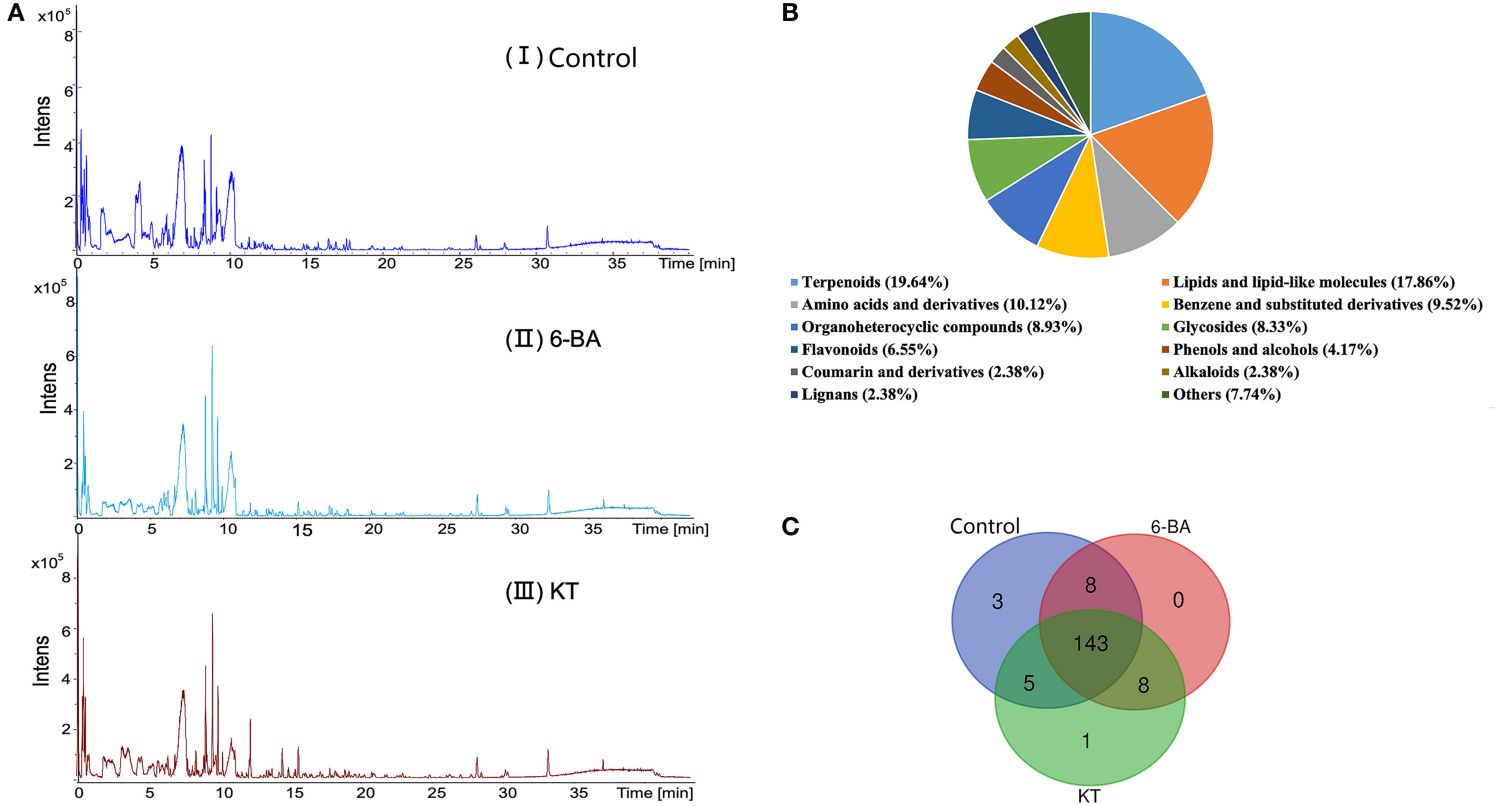
Detection and analysis of metabolites according to UPLC–MS. **(A)** Total ion chromatogram of metabolites in *S. tschiliensis* obtained by UPLC–MS. From top to bottom: (I) Control group, (II) 6-BA group, (III) KT group. **(B)** Classification of 168 metabolites in *S. tschiliensis*. **(C)** Venn diagram from three groups of identified metabolites.

*Scabiosa tschiliensis* with different groups and the typical UPLC–MS spectra are presented in Figure 1A, and detailed information on the detected metabolites is listed in Supplementary Table 2. Eventually, metabolites with 168 were identified or tentatively characterized in *S. tschiliensis*, including terpenoids, lipids and lipid-like molecules, amino acids and derivatives, flavonoids, alkaloids, and lignans, etc. (Figure 1B). Metabolites with 159, 159, and 157 were identified in the control group, 6-BA group, and KT group, respectively. It can be seen from the Venn diagram (Figure 1C) that there are a total of 143 metabolites shared by the three groups, indicating that there is little difference in the types of identified metabolites between the two PGR treatment groups and the control group, and the difference is more reflected in the intensity of metabolite. The three metabolites unique to the control group are physalin D, oleuroside, and plantagoside, which disappeared after adding PGRs, indicating that 6-BA and KT are not conducive to the accumulation of the three metabolites. The 4-methylumbelliferone is a specific metabolite of the KT group, belongs to the class of organic compounds known as coumarins. As can be seen from Figure 1C, eight metabolites are shared by the 6-BA group and KT group, namely, PG (18:0/0:0), umbelliferone, gamma-tocotrienol, (17alpha,23S)-17,23-epoxy-29-hydroxy-27-norlanosta-1,8-diene-3,15,24-trione, oxyallobetulin, na-p-hydroxycoumaroyltryptophan, n-caffeoyltryptophan, and theasapogenol E. These eight metabolites are not present in the control group and only detected after the treatment of PGRs, indicating that 6-BA and KT are beneficial to the accumulation of those metabolites.

### Identification of Key Metabolites Responds to Different PGRs

**Figure 2:**
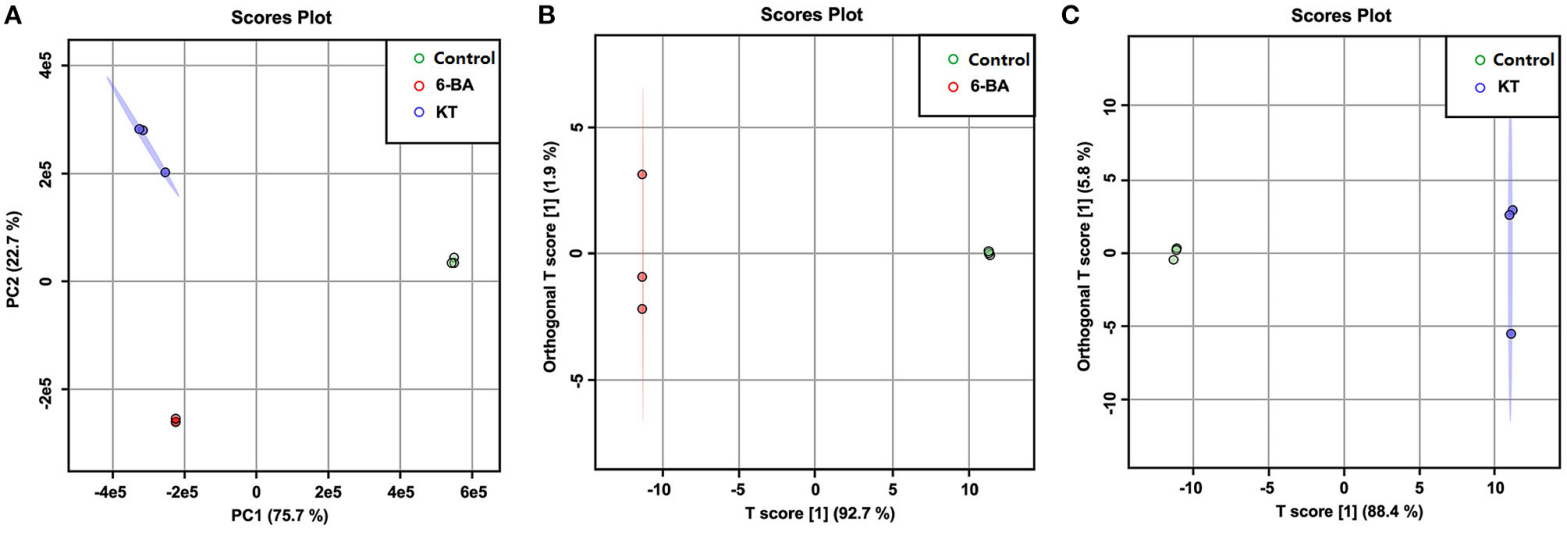
Score plots of models. **(A)** PCA score plots of control, 6-BA, and KT groups. **(B)** OPLS-DA score plots of control and 6-BA group. **(C)** OPLS-DA score plots of control and KT group.

A PCA score plot was constructed based on the UPLC–MS profiling data (Figure 2A), and the result can provide an overall view of *S. tschiliensis* treated with different PGRs. The result of PCA showed that there was a clear discrimination among the samples under different treatments. As shown in Figure 2A, a clear divide between the PGR treatment groups and control group was observed on the PC1 axes (explaining 75.7% of the variation), and the two PGR treatment groups, namely, 6-BA and KT, could be separated on the PC2 axes (explaining 22.7 % of the variation).

Orthogonal partial least-squares discriminant analysis was further used to discover the metabolic influences of the PGRs depending on the UPLC–MS data from control, 6-BA, and KT groups. The scores plot of the 6-BA treatment group (Figure 2B) and the KT treatment group (Figure 2C) indicated a good separation from the control group. According to the corresponding values of *R*^2^ and *Q*^2^ (Supplementary Table 3), and through permutation tests to further perform model-based verification (Supplementary Figure 2), a good prediction and reliable OPLS-DA model was established. In the OPLS-DA, potential differential metabolites are usually screened when variables with variable importance (VIP) >1. And the independent-sample *t*-test (*P*-value) was also calculated to evaluate the statistical significance of the data. Combining the analysis results of the above two, a metabolite was considered to be significantly different based on a VIP > 1 and *P*-value < 0.05, and the results are shown in Supplementary Table 2.

**Figure 3:**
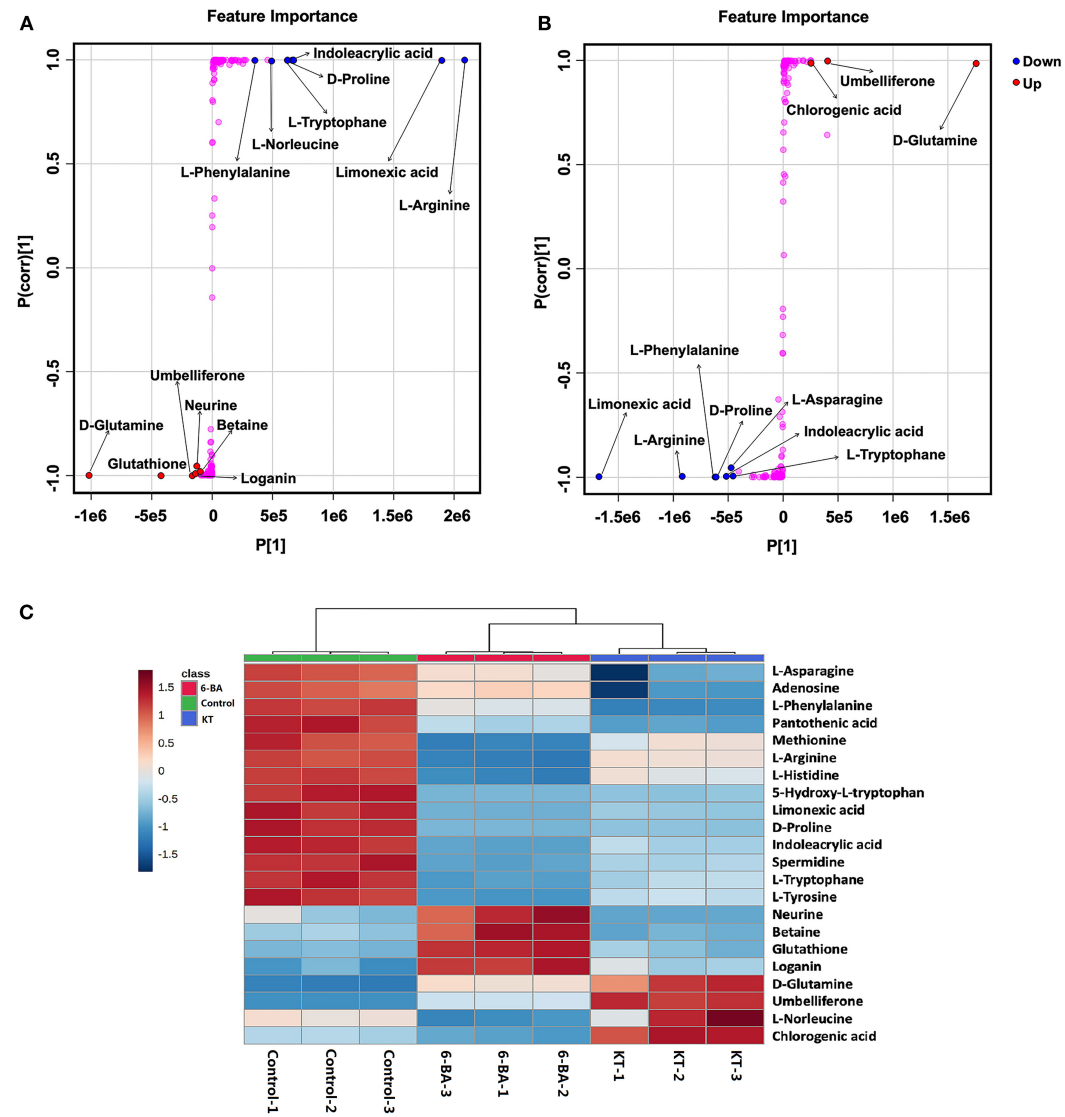
The most important potential differential metabolites. **(A)** Potential differential metabolite identifications from S-plots in the 6-BA group. **(B)** Potential differential metabolite identifications from S-plots in the KT group. **(C)** Heatmap showing the intensities of significant metabolite expression features in PGRs compared with control group.

The most important differential metabolites were screened following the S-plots of OPLS-DA (Figure 3). The S-plot exhibited an appreciable increase in D-glutamine, glutathione, umbelliferone, loganin, neurine, and betaine, while the levels of L-arginine, limonexic acid, indoleacrylic acid, D-proline, L-tryptophan, L-norleucine, and L-phenylalanine dropped significantly in the 6-BA group relative to the control group (Figure 3A). As shown in Figure 3B, the levels of D-glutamine, umbelliferone, and chlorogenic acid increased markedly in the KT group compared with the control group, whereas the intensity of limonexic acid, L-arginine, L-phenylalanine, D-proline, indoleacrylic acid, L-asparagine, and L-tryptophan decreased distinctly.

**Figure 4:**
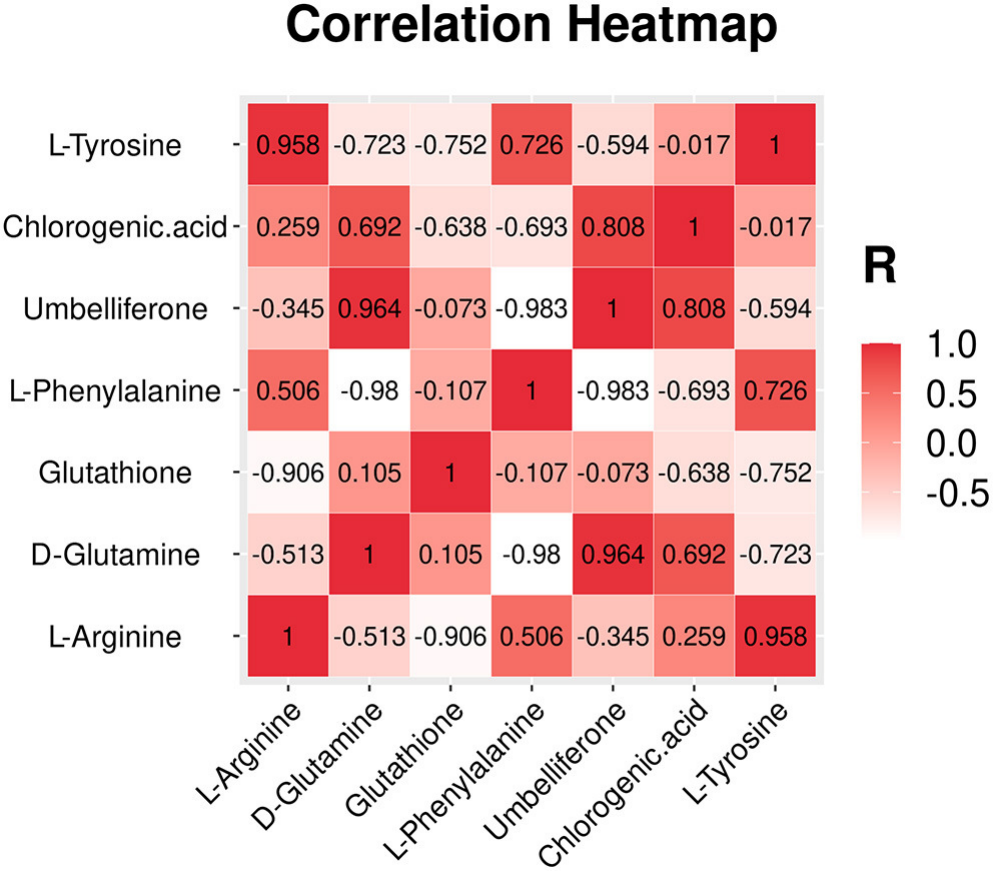
Heatmap based on pairwise Pearson's correlation of significant metabolites.

**Figure 5:**
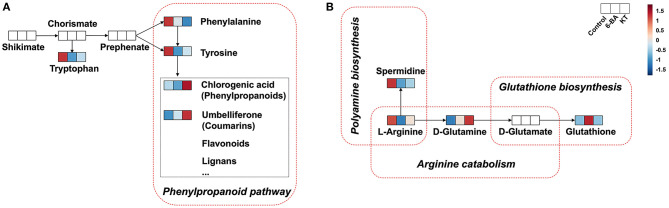
Biosynthesis and catabolism of metabolites. **(A)** Shikimate pathway. **(B)** Arginine catabolism and glutathione biosynthesis (Metabolites not detected in *S. tschiliensis* are indicated in white.).

A heatmap of interested differential metabolites is shown in Figure 3C (MetaboAnalyst), and the specific information is listed in Table 1. Furthermore, the pairwise Pearson linear relationship between significant metabolites is shown by the heatmap (Figure 4), and the bioinformatic analysis was performed using the OmicStudio tools at https://www.omicstudio.cn/tool. The Pearson correlation is −1, which represents a perfect negative linear correlation (Benucci et al., 2011). According to the correlations between the metabolites, the possible pathways of metabolite biosynthesis and catabolism under different PGR effects are shown in Figure 5.

**Table 1:** Regulation of different PGRs on differential metabolites.

**Class**	**Metabolite name**	**6-BA vs. Control**				**KT vs. Control**			
		**VIP**	***P*-value**	**Log_**2**_(FC)**	**Regulated**	**VIP**	***P*-value**	**Log_**2**_(FC)**	**Regulated**
Amino acids and derivatives	L-Arginine	7.02	9.08E-07	−1.80	Down	4.42	1.45E-05	−0.56	Down
	D-Glutamine	4.91	2.10E-06	0.72	Up	6.08	2.83E-04	1.09	Up
	D-Proline	3.94	2.50E-06	−2.97	Down	3.62	3.06E-06	−2.56	Down
	L-Tryptophan	3.82	4.01E-06	−1.47	Down	3.12	4.64E-05	−0.95	Down
	L-Norleucine	3.39	4.22E-05	−0.41	Down	2.35	—	—	—
	L-Phenylalanine	2.87	1.04E-05	−0.54	Down	3.62	9.14E-07	−1.18	Down
	L-Tyrosine	2.30	9.35E-06	−1.63	Down	1.81	5.44E-05	−0.93	Down
	L-Asparagine	2.27	1.56E-04	−0.50	Down	3.11	3.01E-03	−1.51	Down
	Methionine	1.96	2.52E-05	−1.54	Down	1.30	8.42E-04	−0.58	Down
	L-Histidine	1.92	2.64E-06	−1.66	Down	1.32	6.29E-05	−0.65	Down
Alkaloids	Neurine	1.71	3.32E-03	0.33	Up	—	—	—	—
	Betaine	1.52	5.28E-04	0.50	Up	—	—	—	—
Terpenoids	Limonexic acid	6.68	7.15E-06	−2.68	Down	5.97	1.29E-05	−2.08	Down
	Loganin	1.83	8.39E-05	0.36	Up	—	—	—	—
Benzene and substituted derivatives	Chlorogenic acid	1.28	6.49E-04	−0.29	Down	2.30	1.59E-04	0.73	Up
Coumarin and derivatives	Umbelliferone	2.20	2.32E-09	2.33	Up	3.27	8.14E-07	2.37	Up
Organoheterocyclic compounds	Indoleacrylic acid	3.96	1.75E-06	−1.50	Down	3.32	3.06E-05	−1.04	Down
	5-Hydroxy-L-tryptophan	1.25	5.08E-06	−3.71	Down	1.14	6.76E-06	−2.98	Down
Phenols and alcohols	Pantothenic acid	1.23	7.77E-05	−0.61	Down	1.32	1.86E-05	−0.85	Down
Others	Glutathione	3.17	2.21E-06	0.76	Up	—	—	—	—
	Adenosine	1.82	8.09E-04	−0.16	Down	2.89	1.22E-03	−0.51	Down
	Spermidine	1.02	4.26E-06	−2.69	Down	—	—	—	—

*Differential metabolites are screened when variables with variable importance (VIP) > 1 in OPLS-DA analysis and the independent-sample t-test of P-value < 0.05*.

### Annotation Analysis of KEGG of Key Metabolites in Response to Different PGRs

**Figure 6:**
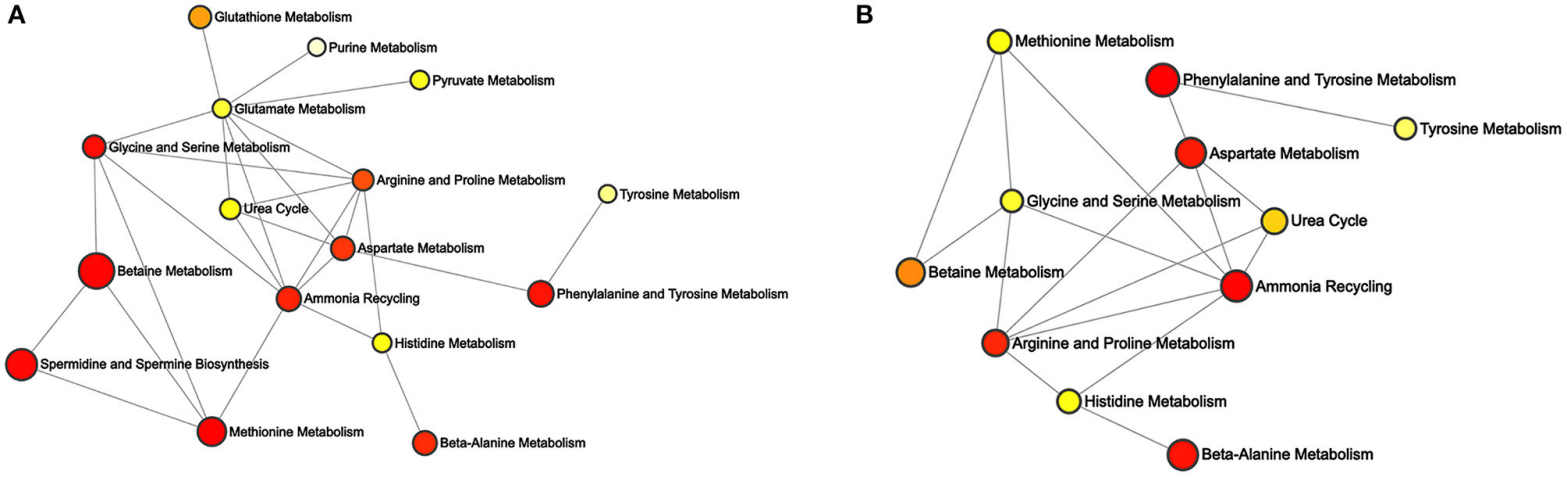
Enrichment analysis of differential metabolites associated with the pathway. **(A)** Differential network between control and 6-BA group. **(B)** Differential network between control and KT group.

Depending on the differential metabolites, enrichment networks were constructed to discover the potential pathways of different PGRs (Figure 6). In these enrichment networks, nodes represent the metabolic pathways, while lines indicate the biological relationships between metabolites associated with the two pathways. Moreover, the *p*-value was exhibited based on the colored ball and the size of the ball according to the multiple enrichment on behalf of the actual matched number (Xia and Wishart, 2011). For the 6-BA treatment group, the major enriched metabolic pathways were betaine metabolism, methionine metabolism, spermidine and spermine biosynthesis, phenylalanine and tyrosine metabolism, and ammonia recycling (Figure 6A). For the KT treatment group, phenylalanine and tyrosine metabolism, ammonia recycling, beta-alanine metabolism, and aspartate metabolism were the main differential metabolic pathways (Figure 6B). The metabolites enriched in a pathway are listed in Supplementary Table 4.

## Discussion

### The Effect of PGRs on Secondary Metabolites

From Figure 3, we can see that neurine, betaine, and loganin were significantly increased in the 6-BA group. In contrast, there were no changes in the KT group, which suggested that 6-BA was beneficial to the accumulation of the three metabolites. Neurine and betaine are alkaloids, which can be biosynthesized from choline. Betaine is believed to have antioxidant properties, and when used as a medicine, it is used to treat gastrointestinal disturbances, liver disorders, homocystinuria, and hyperkalemia (Lever and Slow, 2010; Hassanpour et al., 2020). Loganin is a bioactive compound that belongs to iridoids, which is reported to have anti-inflammatory and neuroprotective effects (Cui et al., 2018). The present results suggest that we can acquire more neurine, betaine, and loganin by adding 6-BA to the *in vitro* propagation of *S. tschiliensis*.

Chlorogenic acid is a very valuable phenylpropanoid, which has anti-inflammatory (Feng et al., 2016) and antioxidant effects (Sato et al., 2011). In recent decades, clinical and scientific researchers have shown that chlorogenic acid intake plays an antihypertensive role (Naveed et al., 2018). We found that KT might be beneficial to the accumulate of chlorogenic acid. Because the chlorogenic acid was found significant increases in the KT treatment group whereas there was a slight decrease in the 6-BA treatment group (Figure 3C).

Umbelliferone belongs to the class of organic compounds known as 7-hydroxycoumarins, which possesses unique biological activity and has been regarded as a drug with antitumor, anti-hyperglycemic, antioxidant, and anti-inflammatory activities (Cruz et al., 2020). Umbelliferone can also induce modifications of cell growth, development and the communication mechanisms of intracellular (Gök, 2013). In this study, umbelliferone did not exist in the control group. Interestingly, it appeared and significantly increased in both PGR treatment groups, which indicated that the addition of 6-BA and KT promoted the biosynthesis of umbelliferone. In addition, KT has a better effect on the accumulation of umbelliferone than 6-BA (Figure 3C).

The present study shows that 6-BA and KT can influence the secondary metabolites differently in *S. tschiliensis*. Moreover, in the cultivation of *Clidemia hirta* L., the production of phenolics, flavonoids, and saponins was influenced by the culture medium (Lopez et al., 2016). We suspect that in the process of tissue culture, there may be some other factors that can also affect the secondary metabolites, which needs further research.

### Biosynthesis Pathway Analysis of Umbelliferone and Chlorogenic Acid

With respect to the metabolic pathways, phenylalanine and tyrosine metabolism is closely related to the addition of 6-BA and KT. In this study, we found that some primary metabolisms in the two PGR groups were decreased, such as L-phenylalanine and L-tyrosine. However, the secondary metabolite umbelliferone in the 6-BA treatment group increased, while the levels of umbelliferone and chlorogenic acid increased in the KT treatment group (Figure 3). In higher plants, the aromatic amino acids produced by the shikimate pathway are not only key components of protein biosynthesis, but also act as precursors for a variety of secondary metabolites essential for plant growth (Tzin and Galili, 2010). The phenylpropanoid pathway is an important part of the shikimate pathway. In the phenylpropanoid pathway (Figure 5A), the principal aromatic phenolic compounds transformed from L-phenylalanine and L-tyrosine are coumarins, flavonoids, lignans, etc. (Santos-Sánchez et al., 2019). The changes in primary and secondary metabolites found in our work are consistent with the metabolite changes in the shikimate pathway, which suggested that the increase in umbelliferone and chlorogenic acid may be biosynthesized from L-phenylalanine and L-tyrosine.

Hitherto, chlorogenic acid biosynthesis has been demonstrated *via* the phenylpropanoid pathway through three different routes in plants (Kundu and Vadassery, 2019). In this study, the correlation coefficient between L-phenylalanine and chlorogenic acid was −0.693 (Figure 4), which indicates that there is a certain negative correlation between the two metabolites. Moreover, we speculate that chlorogenic acid is likely to be biosynthesized through the phenylpropanoid pathway with the help of PGR.

The previous study has proved that the biosynthesis of umbelliferone comes from phenylalanine and tyrosine (Mazimba, 2017). In this study, the Pearson correlation coefficient is −0.983 and −0.594 between umbelliferone with L-phenylalanine and umbelliferone with L-tyrosine, respectively (Figure 4). We can conclude that there is a significant negative correlation between L-phenylalanine and umbelliferone. L -phenylalanine and L-tyrosine are raw materials for the synthesis of umbelliferone, the decrease of the two amino acids in the experiment indicating that umbelliferone is likely to be transformed from the L -phenylalanine and L-tyrosine (Figure 5A). Here, the results of this study indicated that the umbelliferone levels increased obviously in each PGR-treated group, indicating that the *in vitro* propagation technology can be used as a method to provide large amounts of umbelliferone in the future. The specific reason for the presence of umbelliferone remains a further confirmation.

### The Effect of PGRs on Primary Metabolites

Plant regeneration can generally be enhanced by the exogenous supply of plant hormones *in vitro* (Ikeuchi et al., 2016). Cytokinins are an important part of PGR that is related to various physiological processes (Abu-Romman et al., 2015), including cell division, callus induction, shoot initiation and growth, tuberous root production, and leaf senescence (Jeong and Sivanesan, 2015). Two adenine-type cytokinins, 6-BA and KT, differ only in the structure of *N*^6^ substituent. Different structures are possible to influence the role of cytokinins, although the exact functions of the diverse forms are unclear (Kieber and Schaller, 2014). KT is generally used together with low-level auxins to induce shoots or form callus. Meanwhile, 6-BA is normally used to accelerate the growth (Foo et al., 2018).

In this study, 6-BA and KT were used to induce adventitious shoots of *S. tschiliensis*. When cytokinins were applied to a single part of a plant (for example, a leaf), they cause the dealt organ turns into an active pool of amino acids, then amino acids migrate from surrounding parts to the organ (George et al., 2008). This also implies that cytokinin may cause the breakdown of amino acids and then convert them into other metabolites. The phenomenon is consistent with the significant decrease in certain amino acids after the addition of cytokinin in this experiment (Figure 3). At the beginning of the phenylpropanoid pathway, phenylalanine ammonia lyase is one of the important enzymes, which can turn phenylalanine into more phenylpropanoid compounds. And this enzyme can be stimulated by abiotic factors such as cytokinins (Bektaş, 2020). We speculate that under the action of cytokinins, L-phenylalanine and L-tyrosine are decomposed to produce chlorogenic acid and umbelliferone, which further promote the growth and development of cells during adventitious shoot formation. Most differential metabolites showed the same trend by adding 6-BA and KT. However, some of them were different (Supplementary Table 2), which may be caused by the different structures of the two cytokinins. We speculate that other adenine-type cytokinins, such as zeatin riboside and dihydrozeatin, may have similar effects on metabolites in *S. tschiliensis*.

L-Arginine is a unique and significant amino acid in plants. It not only serves as significant nitrogen storage and recycling but also serves as a precursor for the biosynthesis of polyamines, nitric oxide, etc. (Figure 5B). Polyamines and nitric oxide are present in almost all physiological and biochemical processes, which are important messengers for growth and development as well as adaptation of the plant to stress. The arginine metabolism plays an important role in plant perception and adaptation to environmental disturbances (Yang and Gao, 2007; Bokhary et al., 2020). In plant tissues, arginine contents seem to be regulated by multiple mechanisms, since most experimental procedures for arginine biosynthesis or catabolism did not change the concentrations of arginine (Winter et al., 2015). However, we found that the L-arginine showed the same tendency when different PGRs were supplied. Specifically, lower intensity of L-arginine was detected in the 6-BA group and KT group. In this study, L-arginine was enriched in arginine and proline metabolism, aspartate metabolism, glycine and serine metabolism, and urea cycle (Supplementary Table 4). Moreover, these metabolic pathways are closely linked to ammonia recycling (Figure 6). We speculate that the decrease in L-arginine may be caused by a certain reaction under the action of PGRs, and this reaction may be related to ammonia recycling. The previous description of arginine metabolism in higher plants mainly came from studies with Arabidopsis, which found that nitrogen flux from L-arginine to ammonia pools was very large, especially during germination (Winter et al., 2015). The addition of PGRs may create the same environment as during germination, which results in lower L-arginine levels.

After arginine is imported into the mitochondria, arginine catabolism begins to produce glutamate (Winter et al., 2015). As shown in Figure 5B, glutathione can be synthesized from D-glutamate through glutathione biosynthesis. Plants produce glutathione as part of their response to the stress of the environment. Glutathione plays a key role in plant cell proliferation, root development, salt tolerance, resistance to disease, and chilling damage (Galant et al., 2011). In this study, the correlation coefficients between glutathione with L-arginine and that with D-glutamine were −0.906 and −0.513, respectively (Figure 4). We found that the application of 6-BA increased the levels of D-glutamine and glutathione (Figure 5B), and glutathione was enriched in glutamate metabolism and glutathione metabolism (Supplementary Table 4), which illustrates that glutathione was probably derived from L-arginine. In addition, KT primarily increased the level of D-glutamine, and no change was found in glutathione (Figure 5B). We speculate that the decrease in L-arginine is due to the biosynthesis of polyamine or glutathione, and it can ultimately promote plant growth and development. However, the precise derivation process between these metabolites needs further research.

We discovered that the expression of D-proline and L-asparagine was obviously decreased with the treatment of KT relative to the control group. And the level of D-proline decreased markedly, while L-asparagine slightly decreased when 6-BA was added (Figure 3). In the early stages of *in vitro* culture, a medium supplied with hydrolyzed proteins or amino acids was usually used to accelerate the explant proliferation. The previous study has found that proline was a beneficial amino acid, which can stimulate shoot organogenesis from cotyledon explants in *Cucumis melo* (Hamasaki et al., 2005).

Furthermore, L-asparagine and L-proline benefit to the growth of callus and can significantly enhance the number of shoots in sorghum (Liu et al., 2015). In the present study, the decrease in L-asparagine can be considered to provide organic nitrogen supply associated with *in vitro* culture and embryogenesis (Sarker et al., 2007). The accumulation of proline always occurs in response to various environmental stresses in plant (Stein et al., 2011); however, the D-proline decreases with the addition of PGR in this study. We speculate that adding PGR in the tissue culture process will reduce environmental stresses. This causes *S. tschiliensis* to grow in a more appropriate environment, and the decrease in D-proline content may be related to this.

## Conclusions

In summary, the present study established a strategy of using UPLC–MS coupled with the multivariate data analysis method to explore changes in metabolites, which is related to the supplement of PGRs in *S. tschiliensis*. Finally, this paper finds out that amino acids are closely related to the treatment of PGRs, especially those that involve multiple pathways and serve as precursors for diverse secondary metabolites, such as L-phenylalanine, L-tyrosine, and L-arginine. Our findings confirmed that the use of PGRs could affect the production of primary and secondary metabolites in *S. tschiliensis*. Since the amino acid metabolism pathways are important in higher plants, the metabolite changes affected by PGRs will be valued in other plant species. When *S. tschiliensis* is obtained through tissue culture, PGR can be selected according to the required bioactive metabolites. However, based on the results of this experiment, it is recommended to choose 6-BA after comprehensive consideration, because it can not only obtain more kinds of bioactive metabolites but also has a better induction efficiency for adventitious shoots.

## Data Availability Statement

The original contributions presented in the study are included in the article/Supplementary Material, further inquiries can be directed to the corresponding author/s.

## Author Contributions

JW designed the experiments and manuscript. XW developed the UPLC-MS methods. JD performed the metabolomics analysis, interpreted the results, and prepared the manuscript. WM cultivated the experimental materials. WM, YiL, XL, and HH developed the UPLC-MS methods and performed the experiments. ZG, YY, and YuL contributions to the acquisition of data. All authors have read and approved the manuscript for publication.

## Conflict of Interest

The authors declare that the research was conducted in the absence of any commercial or financial relationships that could be construed as a potential conflict of interest.
