# Supplementary material for: UPLC–MS-Based Non-targeted Analysis of Endogenous Metabolite Changes in the Leaves of Scabiosa tschiliensis Grüning Induced by 6-Benzylaminopurine and Kinetin
Source: Front Plant Sci. 2021 Jul 21;12:700623. doi: 10.3389/fpls.2021.700623 (PMC8335593; doi:10.3389/fpls.2021.700623)
Supplement: Supplementary file 1 [file Data_Sheet_1.docx]

**Supplementary Figure Material for:**

**UPLC**–**MS-based Non-targeted Analysis of Endogenous Metabolite Changes in the Leaves of**

***Scabiosa tschiliensis* Grüning Induced by** **6-Benzylaminopurine and Kinetin**

**
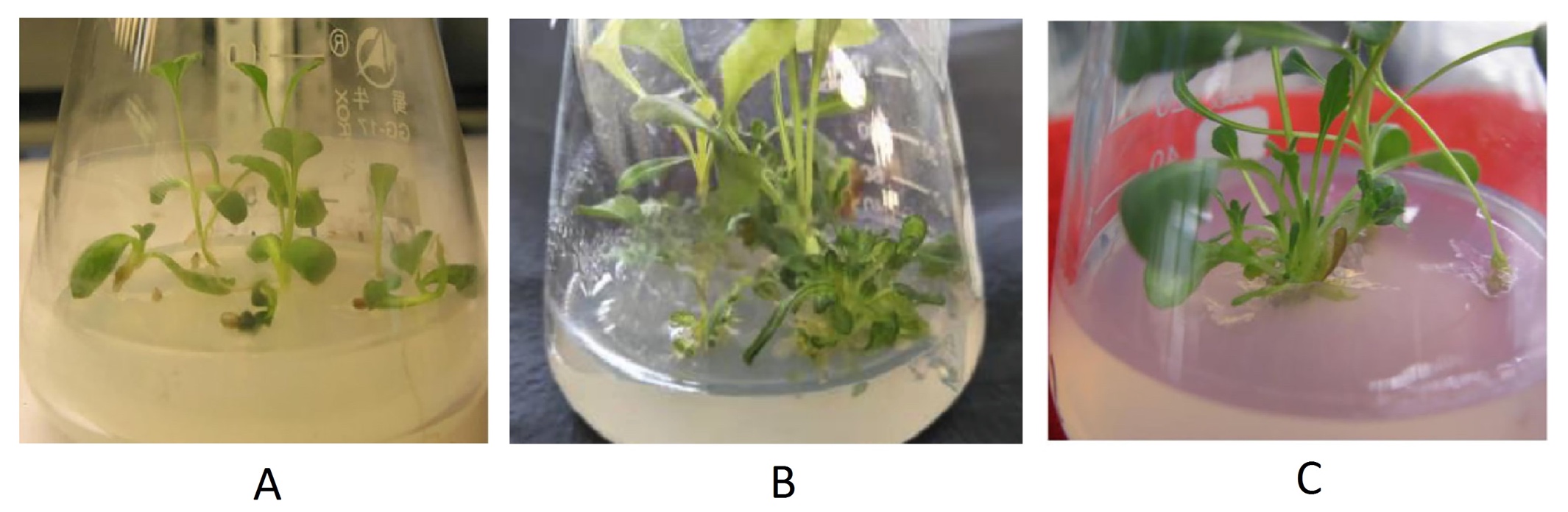
**

**Supplementary Figure 1. Selected leaves of plants with 45 cultivation days as experimental materials.**

A. Shoots from in vitro germinated seeds (control group)

B. Adventitious shoot induced by 6-BA.

C. Adventitious shoot induced by KT.


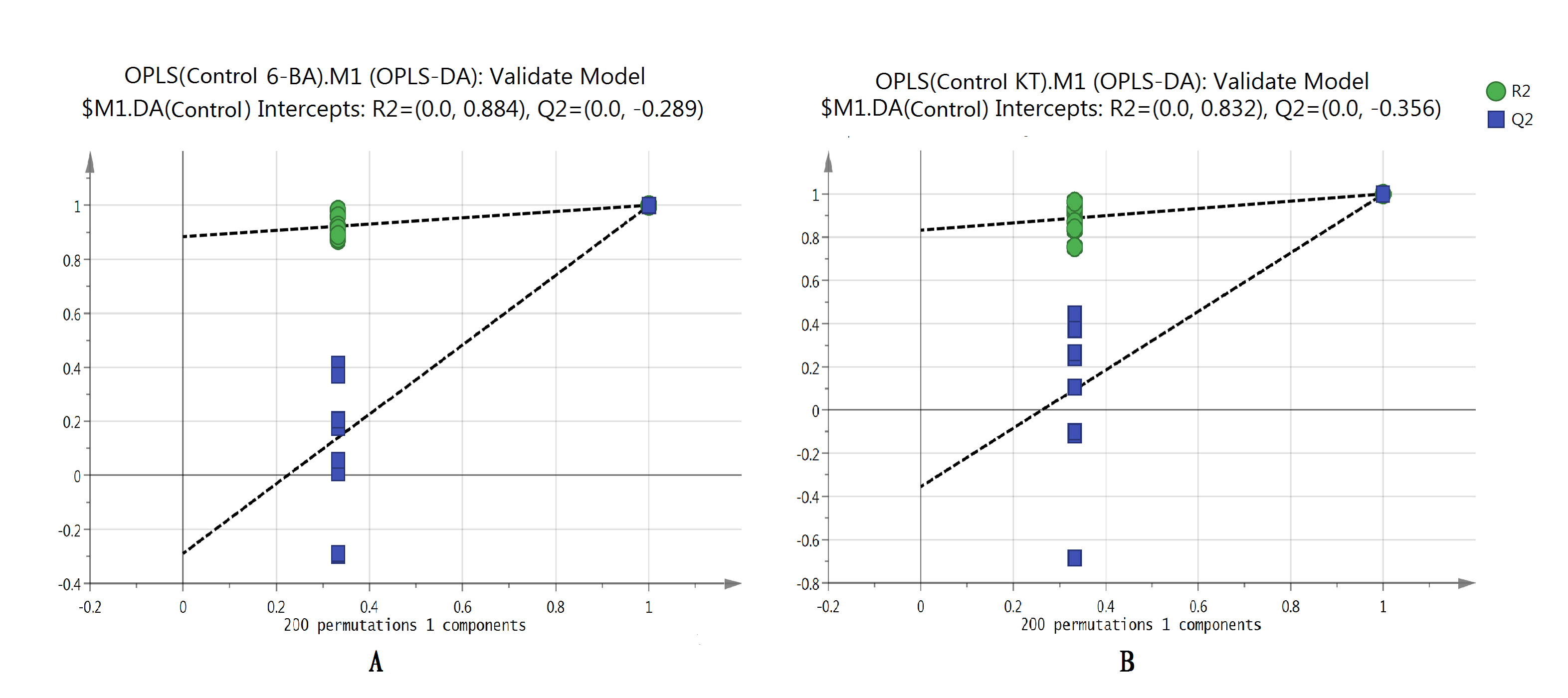


**Supplementary Figure 2. Model-based verification using permutation tests.**

A. Validate model of control and 6-BA group.

B. Validate model control and KT group.
